# Effects of Pictorial Health Warnings on Cognitive, Affective, and Smoking Behavior: A Mixed Methods Study in Four Cities in Indonesia

**DOI:** 10.31557/APJCP.2021.22.2.397

**Published:** 2021-02

**Authors:** Rendro Dhani, Artini Artini, Sri Tunggul Pannindriya, Albert Albert, Abdillah Ahsan, Dian Kusuma

**Affiliations:** 1 *Faculty of Communication Studies, Institut Komunikasi dan Bisnis LSPR, Jakarta, Indonesia. *; 2 *Faculty of Economics and Business, University of Indonesia, Depok, Indonesia. *; 3 *Policy Innovation, Imperial College Business School, London, UK. *

**Keywords:** Tobacco control, smoking behavior, urban, pictorial health warning, Indonesia

## Abstract

While studies have shown the importance of pictorial health warnings (PHW) as a tobacco control strategy, empirical evidence on the efficacy of PHW in prompting smoking behavior remains inconclusive. The study aimed to examine the association between PHW and cognitive reactions, emotional/affective reactions, and smoking behavior. We conducted a mixed-methods study, which included a cross-sectional face-to-face survey of 401 smokers in four cities (Jakarta, Bandung, Semarang, and Yogyakarta) and three focus group discussions among 24 participants in Jakarta. We applied multiple logit regression in STATA for quantitative data analysis and explanatory sequential design for qualitative data analysis. Quantitatively, we found high (63-84% of respondents) understanding about PHW objectives (cognitive reactions), including to remind health risks and encourage smoking cessation. With only 40% PHW, we found relatively low (32%-39%) negative emotional reactions, including feeling scared, annoyed and disgusted and relatively low proportions (33-40%) of respondents that reported quit attempt. Consistent with the quantitative findings, qualitative data provided contexts, including in explaining that the professional worker group was the least affected by PHW, while the student and non-professional groups were the most vulnerable. All this is supportive of governments in Indonesia and other countries to increase the PHW size.

## Introduction

Indonesia, a new upper-middle-income country with a population of 268 million, had an estimated 61.4 million current smokers and over 225 thousand tobacco-related deaths in 2018 (The World Bank, n.d.; World Health Organization [WHO], 2018a). The Indonesian Global Burden of Study also showed that smoking was among the top contributors to a disability, particularly among men in 2017 (Mboi et al., 2018). Male smoking prevalence among adult (15+ years) and youth (13-14 years) was among the highest in the world at 67% (2018) and 36% (2014), respectively (Kusumawardani et al., 2015; WHO, 2018a). The WHO has recommended large PHW, which stipulated in Framework Convention of Tobacco Control, FCTC, Article 11 in order to inform the harmful effects resulting from tobacco use (WHO, 2005). The treaty has now been signed by 168 countries and is legally binding in 181 ratifying countries (WHO, 2017). 

Tobacco control efforts, however, is still lacking in Indonesia partly because the government is reluctant to ratify the FCTC (Kusuma et al., 2019). One flagship national policy was the smoke-free policy started in 2012 that encourages 514 district governments to regulate and ban smoking, advertising, promotion, and selling in selected facility types. Data have shown only two-third of districts adopted the policy locally by 2018 (e.g., Wahidin et al., 2020; Megatsari et al., 2019; Wahyuti et al., 2019). Also, Indonesia has implemented PHW as tobacco control policy since 2014 which requires cigarette companies to cover 40% of the front and back of cigarette packages with five different PHWs. The size of Indonesia’s PHW is among the lowest in the world compared to at least 30%-50% recommended by the FCTC and 65% recommended by the European Union (WHO, 2018b). That is far behind neighboring countries like India and Thailand with PHW 85% of the package. While Timor Leste, a new country once become part of Indonesia, is currently adopting a PHW policy of 92.5% (85% up front, 100% at the back) of their cigarette packs (Canadian Cancer Society, 2018). 

As one of anti-tobacco strategies, large PHW and plain packaging have become a growing worldwide trend. As per August 14, 2019, 107 countries and jurisdictions have adopted the PHW policy with at least 50% of the main display area of the cigarette package, while the PHW with less than 50% was applied only by 16 countries (Canadian Cancer Society, 2018; Cancer Council Victoria, 2019; Tobacco Free Kids, 2019). A new development reports that 13 countries have been running with full implementation of plain packs at retailer level, while 18 other countries have officially adopted laws that require plain packs but are still pending because they are awaiting government determination and other considerations (Tobacco Free Kids, 2020). 

Over the past six decades, there has been a lot of academic research focusing on fear-appeals (Ruiter et al., 2014) including harnessing PHW as a health communication strategy. The appeal of PHW as a research concern is rather linear with the increasing number of countries that have recently adopted large, visible and legible PHWs (Canadian Cancer Society, 2018). Previous studies have shown the effectiveness of PHW on increasing intentions to quit, quit attempts, and smoking cessation, including among adolescence (Brewer et al. 2016; Blanton et al., 2014; Fong et al. 2009; Gendall et al. 2018; Hidayah et al., 2019; Li et al. 2015; Mannocci et al., 2019; Ratih and Susanna, 2018), or even measuring on the smoker’s eye movement (Park et al., 2020). Studies of the use of PHW as an effort to inform about the harmful effects of smoking is also a concern of Indonesian researchers (e.g., Alkaff et al., 2020; Bigwanto, and Soerojo, 2020; Crosby et al., 2019; Fauzi et al., 2017;). 

However, past studies that examining the efficacy of PHW are unimpressive and provide inconclusive evidence (Kuehnle, 2019; Monárrez-Espino et al., 2014). For example, Mutti et al. (2013) revealed that the majority of their respondents would not believe that smoking causes impotence and gangrene. Lee (2018) claimed that PHW did not significantly influence the perceptions of Korean smokers. McQueen and colleagues (2016) obtained that the results were not consistent across all labels and interpretations. More importantly, Monárrez-Espino et al. (2014) noted methodological issues from scholars in examining the effect of PHW on the behavior of smokers. “We found very large heterogeneity across studies, poor or very poor methodological quality” (p. e11). All this evidence raises an interesting research question: Does PHW as a fear arousing strategy really matter to sway smoking behavior? In response to this, the present study focuses on examining the effects of PHW on cognitive, affective, and smoking behavior. We use mixed methods with explanatory sequential design as we believed this research design is the most suitable and valuable methods for examining the issue with a single research question. In doing so, we also try to identify important factors that prevent smokers from quitting.

We are interested in examining PHW and its association to smoking behavior given that cigarette packaging is an important part of the overall tobacco marketing strategy (Germain et al., 2010). The plain package case involving Phillip Morris vs. the Australian government (Knaus, 2017) is strong evidence of how important it is for the company to ensure its brand is clearly visible on cigarette packages. Tobacco companies seriously design cigarette brands, including font types and colors (Bansal-Travers et al.,2011; Dewhirst, 2014) to increase cigarette brand loyalty (Dewes, 2014; Wakefield in al., 2012) and brand awareness in children (Kučerová et al., 2017). In order to fight the cigarette’s persuasion and propaganda, many countries adopting PHW as a fear-appeal strategy. A number of researchers have examined various dimensions of PHW, including PHW testimonial (Brennan et al., 2018; Hammond et al., 2019); and other fear-appeal issues (de Hoog et al., 2007; 2008; Durkin et al., 2018; Morales et al., 2012; Nabi and Myrick, 2018; Tannenbaum. 2015).

**Table 1:** Descriptive Statistics of All-Smoker Sample

	n	%
(a) Characteristics		
Sex		
Male	309	77%
Female	92	23%
Age		
<20 years	63	16%
20 - 29 years	233	58%
30+ years	105	26%
Education		
School	279	70%
University	121	30%
Occupation		
Student	196	49%
Employee	153	38%
Others	52	13%
City		
Jakarta	101	25%
Bandung	100	25%
Semarang	100	25%
Yogyakarta	100	25%
(b) Smoking		
Duration (years)		
0 - 5	155	39%
6-11	122	30%
12-53	124	31%
Monthly spending (IDR)		
10,000 - 160,000	137	34%
161,000 - 450,000	135	34%
460,000 - 3,000,000	129	32%
N	401	

N, sample; %, proportion. For occupation, students include school and university; employees include private companies, civil servants, and entrepreneurs; others include casual workers and unemployed. For education, school includes completed primary and high schools; university includes completed undergraduate and graduate degrees. There was one missing value for education. Minimum monthly wages ranged from IDR 2,000,000 in Yogyakarta city to 4,300,000 in Jakarta in 2019/2020.

**Table 2:** PHW Knowledge, Emotion and Behavior among Smokers in Indonesia 2019

	Proportion	95% CI	
	(%)	Lower	Upper
(a) Cognitive reaction			
I believe that smoking can cause cancer and other serious diseases	76%	72%	80%
I understand PHW aims to remind the dangers of smoking	84%	80%	88%
I understand PHW aims to encourage smoking cessation	70%	66%	75%
I understand PHW also aims for non-smokers to avoid smoking	74%	70%	79%
I understand PHW warning gives more understanding than words only	63%	58%	68%
I am aware of others' rights to be free from smoke, esp. children	88%	85%	91%
(b) Affective emotional reaction			
After looking at PHW in Indonesia (40%), I feel scared	32%	28%	37%
After looking at PHW in Indonesia, I feel disgusted	39%	34%	44%
After looking at PHW in Indonesia, I feel worried about harmful effects	37%	32%	42%
PHW is scary but not enough to stop me from smoking	65%	60%	70%
PHW message is excessive and only to frighten smokers	46%	41%	51%
PHW from other countries (85%) is more disgusting/scary	48%	43%	53%
(c) Current smoking behavior			
I have tried to quit smoking several times but failed	40%	35%	45%
I have refused smoking offers several times	33%	28%	38%
I will try to quit smoking even though current PHW is not large	48%	43%	53%
(d) Future smoking behavior & PHW support			
After looking at PHW (85%), I might reduce spending on smoking	42%	37%	47%
PHW should be 85% to make smokers afraid and stop smoking	40%	36%	45%
I support if government increase PHW size to 80%	44%	39%	49%
N	401		

PHW, Pictorial Health Warning; CI, Confidence Interval; N, sample; %, proportion. The values show proportions of respondents agreeing to the statements on knowledge, emotion, and behavior. We used a Likert scale of strongly agree, agree, neutral, disagree, strongly disagree.

**Table 3:** Association between PHW Knowledge, Emotion and Smoking Behavior in Indonesia, 2019

	(a) Current behavior					
N=401	Tried to quit		Refused offers		Will try to quit	
	Odds Ratio	(SE)	Odds Ratio	(SE)	Odds Ratio	(SE)
(1) Cognitive reaction						
I believe that smoking can cause cancer and other serious diseases	2.83*	(0.78)	3.13*	(0.97)	3.93*	(1.06)
I understand PHW aims to remind the dangers of smoking	2.61*	(0.83)	3.32*	(1.26)	4.25*	(1.38)
I understand PHW aims to encourage smoking cessation	2.40*	(0.58)	3.36*	(0.93)	4.35*	(1.07)
I understand PHW also aims for non-smokers to avoid smoking	1.85*	(0.46)	1.72*	(0.45)	2.37*	(0.57)
I understand PHW gives more understanding than words only	2.11*	(0.47)	3.17*	(0.79)	2.05*	(0.44)
I am aware of others' rights to be free from smoke, esp. children	1.43	(0.48)	2.22*	(0.86)	3.56*	(1.29)
(2) Affective emotional reaction						
After looking at PHW in Indonesia (40%), I feel scared	6.78*	(1.63)	7.05*	(1.70)	7.10*	(1.78)
After looking at PHW in Indonesia, I feel disgusted	3.93*	(0.87)	5.45*	(1.27)	3.06*	(0.66)
After looking at PHW in Indonesia, I feel worried about harmful effects	5.67*	(1.30)	5.74*	(1.34)	5.39*	(1.25)
PHW is scary but not enough to stop me from smoking	2.18*	(0.5)	1.27	(0.29)	1.70*	(0.36)
PHW message is excessive and only to frighten smokers	0.99	(0.21)	0.72	(0.16)	0.9	(0.18)
PHW from other countries (85%) is more disgusting/scary	2.84*	(0.60)	2.84*	(0.64)	3.75*	(0.80)
	(b) Future behavior & support					
	Might reduce spending		PHW should be 85%		Support govt increase PHW	
(1) Cognitive reaction						
I believe that smoking can cause cancer and other serious diseases	3.06*	(0.84)	2.29*	(0.61)	3.58*	(0.99
I understand PHW aims to remind the dangers of smoking	3.47*	(1.16)	3.45*	(1.19)	5.33*	(1.95)
I understand PHW aims to encourage smoking cessation	3.77*	(0.96)	4.71*	(1.27)	3.54*	(0.88)
I understand PHW also aims for non-smokers to avoid smoking	2.66*	(0.68)	2.59*	(0.67)	2.45*	(0.61)
I understand PHW gives more understanding than words only	4.62*	(1.11)	3.76*	(0.89)	3.22*	(0.73)
I am aware of others' rights to be free from smoke, esp. children	2.53*	(0.92	3.14*	(1.22)	3.70*	(1.43)
(2) Affective emotional reaction						
After looking at PHW in Indonesia (40%), I feel scared	7.09*	(1.72)	6.72*	(1.62)	6.02*	(1.44)
After looking at PHW in Indonesia, I feel disgusted	4.23*	(0.94)	5.14*	(1.16)	4.27*	(0.95)
After looking at PHW in Indonesia, I feel worried about harmful effects	6.19*	(1.43)	4.74*	(1.07)	5.18*	(1.17)
PHW is scary but not enough to stop me from smoking	1.51	(0.33)	3.39*	(0.82)	2.34*	(0.52)
PHW message is excessive and only to frighten smokers	0.79	(0.16)	0.86	(0.18)	0.92	(0.19)
PHW from other countries (85%) is more disgusting/scary	3.43*	(0.73)	5.69*	(1.29)	4.52*	(0.98)

N, Sample; PHW, Pictorial Health Warning; SE, Standard Error; Odds ratios were from logit regressions of smoking behavior on knowledge and emotion, controlling for sex, age, and education (in STATA 15.1). * = significant at 5% level.

**Figure 1:**
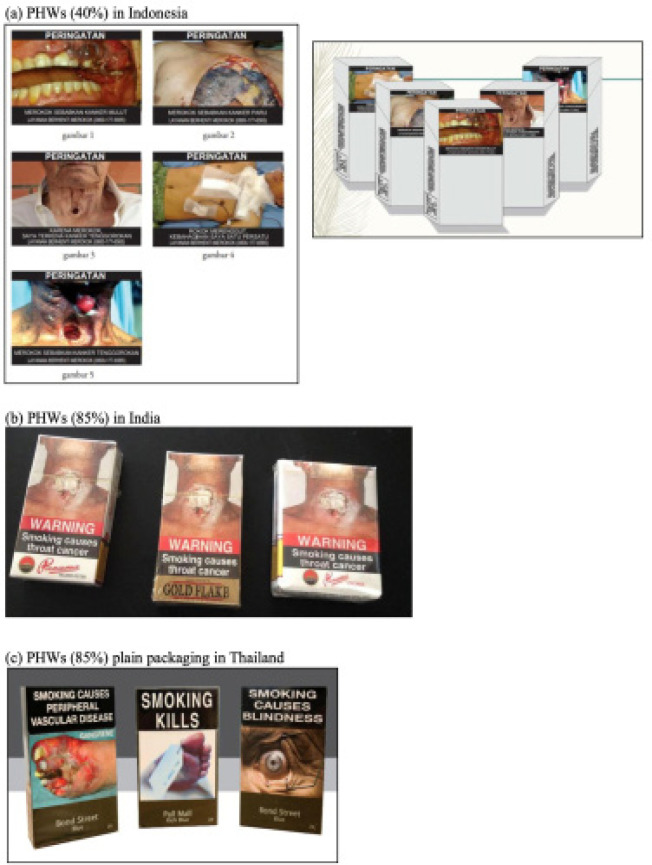
Sample of PHWs Currently Used in Indonesia, India, and Thailand

## Materials and Methods

We used a mixed methods approach to combine the strengths available in qualitative and quantitative research and minimize the limitations of both approaches (Creswell, 2018; Schulze, 2003). We employed explanatory sequential of mixed methods design, by gathering quantitative data and analyzing it first, then qualitative data is collected and analyzed (Shorten and Smith, 2017). Quantitatively, we looked for relationships between smokers’ knowledge and emotions that can influence their decision to quit, reduce, or keep smoking. Qualitatively, we explored smokers’ insights, experiences, and factors that influence their attitudes and behavior. Ethical clearance was obtained from Hasanuddin University Faculty of Public Health, Makassar.

For the quantitative approach, we conducted a cross-sectional survey among 401 smokers in four major cities including Jakarta (101 respondents), Bandung (100), Semarang (100), and Yogyakarta (100). The inclusion criteria included male and female, age of 13+ years old, currently smoking, and willingness to participate. Our questionnaire was adapted from the Indonesian version of the Global Adults Tobacco Survey (GATS) questions translated and validated by Ministry of Health. Our questionnaire includes questions on sociodemographic (e.g. gender, age, occupation, education) as well as on cognitive assessment and emotional reactions to PHW and smoking behavior. Data collection was conducted in-person by eight trained enumerators (two in each city) through face-to-face paper-based interviews during October-November 2019. We used a five-point Likert scale from strongly agree, agree, neutral, disagree, and strongly disagree. Data collection targeted two facility types including educational institutions (e.g. schools and universities) and public places (e.g. malls, kiosks, traditional markets, parking areas, mosques, and offices). In terms of analysis, we provided descriptive analysis on the proportion of cognitive and emotional reactions to PHW and smoking behavior. We also estimated odds ratios for their associations using multiple logit regressions, controlling for sex, age, and education. We used statistically significant level of 5%. Dependent variables include current smoking behavior (e.g. attempted to quit, refused smoking offers, and will try to quit) as well as future behavior and support (e.g. might reduce spending on cigarette, support that PHW should be 85% of cigarette package, and support for government to increase PHW size). We conducted the analyses in STATA 15.

For the qualitative method, we conducted three focus group discussions (FGDs) in Jakarta as the most populous and diverse setting. We purposively selected with 24 participants (eight in each group) with professional background (i.e. higher education and income level), non-professional background (i.e. lower education and income level), and students. Participants age ranged from 16 to 58 years old. The focus groups were conducted as semi-structured interviews. The discussions were recorded and later transcribed into Microsoft Word. We ask questions by referring to three central themes according to the survey questionnaires to explore smokers’ awareness, emotions, and behavior towards 40% PHW on cigarette packs. Before each FGD, we explained the purpose of the study, asked the participants’ consent and showed them some sample packs of cigarettes sold in Indonesia with 40% PHW and also printed images of cigarette packs from India and Thailand with 85% PHW that we used as a comparison (Figure 1). Data collection were conducted face-to-face by six trained interviewers during November-December 2019. In terms of analysis, we performed qualitative data analysis using thematic analysis techniques to explore themes related to cognitive and emotional reactions to PHW and smoking behavior. Both quantitative and qualitative data are analyzed independently to see whether they yield the same results or not.

## Results

Table 1 shows descriptive statistics of our sample who are all smokers. In terms of characteristics (panel a), we analyzed a total of 401 individuals, including 309 (77%) males and 92 (23%) females. Sixteen percent of sample were 13-19 years old, 58% were 20-29 years old, and 26% were 30-68 years old. Among the sample, 70% had completed primary and junior/senior high schools while 30% had completed undergraduate/postgraduate degree. Forty-nine percent of sample were students, 38% were employees and entrepreneurs, and 13% were casual workers or unemployed. Referring to smoking behavior (panel b), 39%, 30%, and 31% of respondents have been smoking for 0-5 years, 6-11 years, and 12-53 years, respectively. Moreover, 34%, 34%, and 32% of sample had monthly cigarette spending of IDR 10,000-160,000, IDR 161,000-450,000, and IDR 460,000-3,000,000, respectively. For perspective, minimum monthly wages in the four cities ranged from IDR 2,000,000 in Yogyakarta city to 4,300,000 in Jakarta in 2019-2020.

*Quantitative Findings*

Table 2 shows the cognitive reactions and affective emotional reactions to PHW and smoking behavior. On cognitive reactions (panel a), 76% of sample believed that smoking can cause cancer and other serious diseases. Also, 70% to 84% of respondents agreed PHW is to remind the danger of smoking, to encourage cessation, and to avoid smoking among non-smokers. Sixty-three percent of respondents agreed that PHW gives more understanding of health risks than note warning only. Moreover, 88% of respondents were aware of others’ rights (especially children) to be smoke free. 

On emotional reactions (panel b), 32% to 39% of sample felt scared, disgusted, and worried about the harmful effects. And 65% agreed that PHW is scary but not enough to stop themselves from smoking. Also, 46% of sample agreed that PHW message is excessive and only to frighten smokers and 48% agreed that PHW from other countries (85%) is more disgusting and scarier. On smoking behavior (panels c-d), 40% of sample said they have tried to quit several times but failed while 33% said they have refused smoking offers. And 48% agreed to try to quit even though the current PHW is not large compared to other countries while 42% said that after looking at larger PHW from other countries, they might reduce spending on smoking. Up to 44% of sample agreed and were supportive for larger PHW size in Indonesia. 

Table 3 shows the association between cognitive and emotional reactions to PHW and smoking behavior. In terms of cognitive reactions, belief that smoking can cause cancer, understanding of PHW objectives (e.g. to remind the danger of smoking, to encourage cessation, and to prevent smoking), and awareness of others’ rights to be smoke free are associated with higher odds of trying to quit (Odds Ratio [OR], up to 2.83), refusing smoking offers (OR up to 3.36), will try to quit (OR up to 4.35), and plan to reduce spending (OR up to 4.62). Moreover, those cognitive reactions are also associated with higher odds of support that PHW should be 85% (OR up to 4.71) and that the government to increase PHW size (OR up to 5.33). All estimates, but one, are significant at 5% level. 

In terms of emotion reactions, feelings of scared, disgusted, and worried about the danger of smoking from PHW in Indonesia (40% of package) and other countries (85% of package) are associated with higher odds of trying to quit (OR up to 6.78), refusing smoking offers (OR up to 7.05), will try to quit (OR up to 7.10), and plan to reduce spending (OR up to 7.09). They are also associated with higher odds of support that PHW should be 85% (OR up to 6.72) and that the government to increase PHW size (OR up to 6.02). All estimates are significant at 5% level. 

*Qualitative Findings*

In terms of cognitive reactions, we basically found no differences compared to the survey results, especially on the knowledge of the harmful effects of cigarettes and other consequences of smoking on human health. The majority of participants in three different groups was aware and understood how smoking can cause negative health effects. Participants in all three groups did not even ask about the truth or validity of the images used as health warnings, except one participant who asked, “what is the ingredient of cigarette that could make the lungs moldy?” (male, non-professional group, 40 years old).

In terms of emotional reactions, we obtained different reactions among the three discussion groups. In the professional group, we found a minority of participants admit that they are quite disturbed with PHW, even though their emotion did not reach to the level of scary. Meanwhile, the majority of participants in this group stated that PHW, both in 40% and 85%, did not scary and disgust them. Some participants agreed that they only felt a little annoyed with PHW but it happened at the beginning when the policy began to be applied to all cigarette packs. “Initially, it affected my emotions but seemed ordinary packaging a few months later” (male, professional group, 43 years old).

However, in the other two groups (student and non-professional) admitted that every time they buy cigarettes they can see the warning pictures and they feel either disturbed, disgusted, or afraid or a combination of these feelings. The emotional reactions of participants included: “Frankly, these warning images (40%) have frightened me, but because the price of cigarettes is cheap, the fear is reduced” (male, non-professional, 48 years old). “I am really scared of the danger of smoking because PHW shows the effects that can cause death” (male, student group, 16 years old). The emotional reactions of participants in these two groups increased even more when we showed them large PHWs (85%). It was reflected both in their spontaneous comments and non-verbal communication. Participants, among others, said: “If the warning pictures on cigarettes are enlarged, then the picture will continue to overshadow me because the consequences are very clear” (male, non-professional, 48 years old). 

In terms of smoking behavior, we found a number of statements that indicate the attitudes and behavior of participants. Some of them noted salient factors that may drive his behavior change. A male participant from the professional group, for example, stated that PHW can be more influential if the policy is combined with other anti-smoking strategies, such as free smoke area policy. Participants said: “For me, the most important factor is the cigarette’s price, because if you ask me whether or not the warning picture has an impact, the answer is no” (female, professional group, 45 years old). “I am sure I will stop smoking completely ... I am afraid of dying just because of cigarettes” (male, non-professional group, 58 years old). One participant from a group of students showed their intention to stop smoking, but experienced difficulties due to the peer group. “I understand and afraid the dangers of smoking, but I saw my environment, my friends, including my teachers, some of whom also smoked. It was hard to get out of a friend’s gang because we were in the same school” (male, student group, 16 years old).

In addition, all participants from the three groups seemed to have comparable perspectives and attitudes in addressing questions, especially in expressing their support if the government implemented an 85% PHW policy. Participants, among others, said: “I completely support the larger PHW policy” (male, non-professional group, 40 years old). “Proceed, if the government wants to increase its size” (male, professional group, 43 years). One participant suggested that larger PHWs would be acceptable because the policy was corresponding for novice smokers, adolescence, and non-smokers. He said: “I agree because it might break the chain of smokers. At least we have to protect our young generation with PHW” (male, professional group, 45 years).

## Discussion

Our findings showed relatively high (63-84%) understanding (cognitive reactions) about PHW objectives including to remind health risks, encourage smoking cessation, or avoid smoking for non-smokers. However, we found relatively low (32%-39%) negative emotional reactions including feeling scared/disgusted and relatively low proportions (33-40%) of respondents that reported quit attempt. Moreover, our study found those who positive cognitive reactions (e.g. believe in health risks and understood PHW objectives) are up to five times more likely to try to quit, refuse smoking offers, plan to reduce spending, and to support government to increase PHW size. Similarly, those who had negative emotional reactions are up to seven times more likely to do so. This result was in line with our qualitative findings, in which the majority of participants have understood and also believed in the health messages conveyed by PHW.

While the relatively high understanding (positive cognitive reactions) is potentially due to the fact that PHW has been implemented since 2014, the relatively low negative emotional reactions may be due to the smaller PHW size in Indonesia (40% of the pack). Furthermore, the qualitative results showed that professional worker group was least emotionally affected by PHW, while students and non-professional groups are most vulnerable to PHW influence, especially when the PHW size is enlarged. In the professional group, one participant claimed to be afraid and another participant who felt annoyed at PHW. However, in the non-professional and student groups, one third of participants reported negative emotions to PHW. 

All countries that have shown PHW effectiveness (e.g. stronger belief about health risks, strong negative emotional reactions, and increased quit attempts) have had larger PHW size including 80% in Sri Lanka and Uruguay, 82.5% in Australia, 85% in India and Thailand, 87.5% in New Zealand, and 90% in Nepal (Brewer et al., 2016; Canadian Cancer Society, 2018; Fong et al., 2009; Gendall et al., 2018; Li et al., 2015; Ratih and Susanna 2018; WHO, 2018; World Health Organization, 2019;). Also, the low proportions of respondents with negative emotional reactions may also due to warning wear-out, as mentioned by one of our focus group discussion participants. A study on the longer-term impact of PHW in Australia, the United Kingdom, and Canada showed that PHW size is an important factor in preventing warning wear-out (Li et al., 2015). 

Furthermore, the relatively low negative emotions and quit attempt due to the smaller PHW size might also due to the counter efforts by the tobacco industry. A study of cigarette pack reviewing tobacco company documents showed that cigarette pack design is an important communication device for cigarette brands and acts as an advertising medium. Leaving cigarette brands on the packaging also allows tobacco companies to market their products by creating a significant in-store presence at the point of purchase and communicating brand image (Wakefield et al., 2002). In Indonesia, a study of cigarette packs shows that cigarette companies harnessed the remaining space of cigarette packs (60%) to display their catchy brands and to advertise certain events or achievements in a fancy way (Bigwanto, and Soerojo, 2020). 

In November 2019, the government has started discussion to increase PHW to 90% of the pack but already received rejections mainly from the cigarette company association (Anwar, 2019). 

Our findings are highly relevant for policy for at least two reasons. First, this provides evidence for the government to increase the PHW size to at least 80% to be more effective in encouraging smoking cessation among smokers and preventing smoking particularly among youth. Larger PHW size would further improve understanding (positive cognitive reactions) on the PHW objectives. It would also increase negative emotional reactions, which in effect would increase smoking cessation and prevention, especially among youth. Secondly, our findings provide evidence to improve the current PHW policy to complement other tobacco control efforts in the country, including the smoke-free policy and outdoor tobacco advertising ban in selected districts (Wahidin et al. 2019; Nurjanah et al. 2019; Megatsari et al. 2019; Wahyuti et al. 2019). All this is crucial to prevent smoking among youth in a country with high peer pressure (‘If I don’t smoke, I’m not a real man’) and cigarette advertising (Nawi et al. 2007; Prabandari and Dewi 2016).

Our study has at least two limitations. First, our research was only among smokers, which did not inform the cognitive and emotional reaction among non-smokers. Second, due to limited resources our study had smaller sample for each city, which did not allow for subgroup analyses to explore variations among city with regard to cognitive and emotional reactions to PHW and smoking behavior. Despite all this, our findings have important policy implications for Indonesia and other countries.

In conclusion, this study offers new evidence on the effects of PHW on cognitive, affective, and smoking behavior using a mixed methods study in four cities in Indonesia. We found relatively high (63-84%) understanding about PHW objectives (e.g. to remind health risks, encourage smoking cessation), but relatively low (32%-39%) negative emotional reactions (e.g. feeling scared/disgusted) and low (33-40%) quit attempts. The qualitative results showed that professional worker group was least emotionally affected by PHW, while students and non-professional groups are most vulnerable to PHW influence, especially when the PHW size is enlarged. Those who understood PHW objectives are up to five times and those who had strong negative emotional reactions are up to seven times more likely to try to quit, refuse smoking offers, plan to reduce spending, and to support government to increase PHW size. Given the support for larger size and more frightening images as shown in other countries, PHW is potential for tobacco control policy in Indonesia and beyond to encourage smoking cessation and to prevent smoking including among youth. 
